# Association between Exposure to Volatile Organic Compounds and the Prevalence of Sleep Problems in US Adults

**DOI:** 10.3390/toxics12030222

**Published:** 2024-03-18

**Authors:** Jianyun Sun, Chunyan Gui, Ya Xiao, Runxue Ma, Ce Liu, Li He, Hao Zhao, Bin Luo

**Affiliations:** 1 Gansu Provincial Centre for Diseases Prevention and Control, Lanzhou 730000, China; 2 Institute of Occupational Health and Environmental Health, School of Public Health, Lanzhou University, Lanzhou 730000, Chinaliuc2017@lzu.edu.cn (C.L.); hel2021@lzu.edu.cn (L.H.);

**Keywords:** volatile organic compounds, short sleep duration, sleep problems

## Abstract

Background: While mounting evidence suggests a connection between environmental contaminants and sleep problems, it remains uncertain whether exposure to volatile organic compounds (VOCs) specifically is associated with such problems. Methods: Data from the National Health and Nutrition Examination Survey program’s five survey cycles (2005–2006, 2011–2018) were used to conduct cross-sectional research. Data on short sleep duration (SSD) and self-reported trouble sleeping were collected from questionnaire data. Data on urine VOCs were gathered from laboratory data. The association between urinary VOCs and sleep problems was examined using weighted generalized linear models and the restricted cubic spline (RCS), weighted quantile sum (WQS), and quantile-based g-calculation (QGC) methods. Results: In all, a total of 4131 general adult individuals were included in this study. The prevalence of SSD and self-reported trouble sleeping was 34.11% and 25.03%, respectively. 3,4-MHA, AAMA, AMCC, SBMA, and MA were risk factors for SSD after adjusting several covariates, with the largest effect being AMCC (OR = 1.47, 95% CI: 1.08, 2.02). Risk factors for sleep issues included AAMA, AMCC, CEMA, CYMA, DGBMA, 2HPMA, 3HPMA, MA, and PGA, with AMCC having the highest impact with an OR of 1.69 (95% CI: 1.28, 2.22). Both the WQS model and the QGC model showed that the co-exposure to VOCs was positively associated with SSD and self-reported trouble sleeping, with AMCC being the most influential VOC. Conclusions: According to our research, high levels of single or mixed urine VOCs are linked to a higher prevalence of SSD and self-reported trouble sleeping in the general adult population of the United States. Further prospective and experimental studies are needed in the future to validate these potential relationships and explore the underlying mechanisms.

## 1. Introduction

Sleep, constituting a significant portion of human life, is a vital and distinctive functional state of the brain with profound implications for public health and economic well-being [1] A good night’s sleep extends beyond merely reducing fatigue and sleepiness, encompassing positive effects on cardiovascular health, metabolic function, immune response, cognitive abilities, and emotional well-being [2]. However, with the accelerated pace of life and increased pressure, people’s sleep time and sleep quality are greatly affected. Sleep problems have 7.6% overall prevalence, according to data analyzed from a survey carried out in 70 countries [3]. According to the findings of a national survey, Australian adults frequently experience sleep disorders [4]. The age-adjusted prevalence of sleep difficulties in the US general population trended upward from 2005 to 2018 [5]. Sleep problems, especially sleep deprivation, are risk factors for obesity, diabetes, cardiovascular disease, dementia, anxiety and depression, declined fertility, and cancer, as well as increased risk of death [6,7,8]. Sleep problems have become a public health issue that cannot be ignored, and it is necessary to explore the risk factors for sleep problems.

A multitude of factors influence sleep, including lifestyle, environmental circumstances, psychological state, health problems, and drug use [9]. Recently, attention has been drawn to environmental risk factors for sleep problems, and several recent studies have reported associations between environmental pollutants and sleep problems. Short-term environmental pollution is associated with sleep disorders in Chinese older individuals, according to Tang et al. [10]. Short sleep duration (SSD) during adolescence has been associated with higher urine quantities of phthalate metabolites [11]. Organophosphorus pesticide exposure is associated with a higher prevalence of SSD in the general adult population in the United States [12]. Chronic pesticide exposure is associated with sleep disorders [13]. Many studies have found higher-than-allowable volatile organic compound (VOC) concentrations in bedrooms during sleep due to poor ventilation, which might cause sleep problems [14,15].

VOCs are common and complex organic pollutants in the air, and the most common groups of VOCs include alkanes, olefins, aromatics, halocarbons, and carbonyls [16]. Many industrial activities, smokestacks, exhaust emissions from cars and other types of vehicles, the incineration of waste and combustion systems, fuels, chemical manufacturing processes, and more are among the main sources of VOCs [17]. In addition, volatile organic compounds are also produced by metabolic reactions in the human body. VOCs can affect the human respiratory system, nervous system, digestive system, urinary system, hematopoietic system, endocrine system, and immune system in a variety of ways such as inflammatory response and oxidative stress, and they may even lead to cancer [18,19]. According to Zhuang et al., benzene and ethylbenzene are positively correlated with depression [20]. Adolescent male rats exposed to benzene and m-xylene showed impaired learning capacity, motor impairments, and anxiety-like behavior [21]. Increased benzene concentrations were associated with an increased incidence of central nervous system symptoms in residents of Gulf countries [22]. Although the neurological risks of VOCs have been extensively studied, research is still needed on the relationship between urinary VOCs and sleep problems.

Therefore, the present study was conducted to investigate the relationship between urinary VOC metabolites and sleep problems using the weighted generalized logistic model, restricted cubic spline (RCS), the weighted quantile sum (WQS) model, and the quantile-based g-calculation (QGC) method using data from the National Health and Nutrition Examination Survey (NHANES).

## 2. Materials and Methods

### 2.1. Study Data

The National Center for Health Statistics (NCHS) of the Centers for Disease Control and Prevention conducts the cross-sectional NHANES to assess the health and nutrition status of Americans. The NHANES runs on a two-year cycle, with all participants providing informed consent. The NHANES was approved by the NCHS Research Ethics Review Committee.

All related data were downloaded from https://www.cdc.gov/nchs/nhanes/index.htm (accessed on 20 August 2023). Because only five cycles (2005–2006, 2011–2012, 2013–2014, 2015–2016, and 2017–2018) measured the amount of VOC metabolites in urine, we used these five cycles of publicly accessible data from the NHANES for this investigation. The NHANES randomly selected a sample of one-third of the population to assess their urine VOC metabolites. Throughout the five cycles, 48,933 participants signed up for the NHANES, of which, 12,435 complete results were obtained from the testing of urine VOC metabolites. Of these, 4227 participants < 20 years of age were excluded from the analysis. After excluding individuals who lacked results from the Sleep Problems Questionnaire (n = 38) and those who lacked data on other covariates (n = 4039), 4131 individuals were ultimately enrolled in the study for analyses of the association between urinary VOCs and sleep problems in US adults (Figure S1).

### 2.2. Exposure Ascertainment

Participants’ urine was collected using a sterile collector by trained professionals and stored in a polypropylene centrifuge tube or polystyrene cryotube vial, before which, participants did not need to fast or eat a special diet. Urine samples were transported at −20 °C and subsequently frozen at −70 °C until analysis. The detection of VOC metabolites in human urine was achieved using ultra-high-performance liquid chromatography–electrospray tandem mass spectrometry (UPLC-ESI/MSMS). Please visit the webpage at https://wwwn.cdc.gov/nchs/data/nhanes/2015-2016/labmethods/UVOC_UVOCS_I_MET.pdf (accessed on 20 August 2023) for more information about laboratory techniques for the detection of urine volatile organic compounds.

Throughout five cycles, the NHANES detected 26 different VOC metabolites in urine. They adjusted values below the lower limit of detection (LOD) to correction values (LOD/sqrt [2]) based on laboratory documentation. We removed 11 VOC metabolites because these metabolites accounted for more than one-third of the correction value [23]. Finally, the analysis comprised 15 urine VOC metabolites in total (Table 1).

### 2.3. Outcome Ascertainment

SLQ050 in the NHANES questionnaire was as follows: Ever told doctor had trouble sleeping? Those who answered yes were considered to have self-reported sleep problems. The questionnaire data for SLD012 (sleep hours—weekdays or workdays) were used to determine whether participants had SSD. The National Institutes of Health advised adults to sleep 7–8 h every day, with SSD being defined as getting less than 7 h of sleep [24].

### 2.4. Covariates

Age, gender, race, education level, marital status, physical activity, drinking alcohol, smoking, household income to poverty level (PIR), body mass index (BMI), and urinary creatinine were all gathered and set as covariates for statistical analysis. Based on the grouping of NHANES questionnaire data, race information included Mexican American, other Hispanic, non-Hispanic white, non-Hispanic black, and other races. Education levels were classified as less than 9th grade, 9th–11th grade, high school graduate/GED or equivalent, some college or AA degree, and college graduate or above. Marital status was classified as married, widowed, divorced, separated, never married, and living with a partner. Physical activity was categorized as never, moderately physically active, and strenuously physically active. Based on previous research, activities that cause a small amount of sweating or a moderate increase in breathing or heart rate are considered to be moderately physically active [25]. Activities that caused significant increases in respiration or heart rate or excessive sweating were considered to be strenuously physically active [25]. Serum cotinine was used to adjust for participant tobacco exposur [20]. Alcohol consumption was classified as having more than 12 drinks per year (yes) or less than 12 drinks per year (no).

### 2.5. Statistical Analysis

Continuous variables are described as weighted means ± standard error, and categorical variables are described as percentages. Urinary VOC metabolites were described as median (quartiles) and were log-transformed with a base of 10 in subsequent analyses to improve normality. Differences between groups were explored using *t*-tests, chi-square tests, or Wilcoxon rank-sum tests. Weighted generalized linear models were applied to estimate the association between urinary VOC metabolites and SSD and sleep difficulties. Odds ratios (ORs) and 95% confidence intervals (CIs) were used to describe these results. Model 1 did not adjust for other factors. Model 2 adjusted for creatinine, sex, age, educational level, race, marriage, PIR, BMI, serum cotinine, drinking alcohol, and physical activity.

Using a restricted cubic spline (RCS) that was adjusted for the aforementioned covariates, it was possible to determine potential nonlinear correlations between dose–response associations between VOC metabolites and sleep problems. Using weighted quantile sum (WQS) regression analysis and quantile-based g calculation (QGC) modeling, we evaluated the influence of co-exposure to VOCs on sleep in order to quantify the total effect of the 15 VOC metabolites on sleep problems as well as the contribution of each VOC. However, the limitation of the WQS model is that it believes that the exposure between multiple mixtures is additive and that the association between exposure and outcome is consistent and linear [26]. So, we also used the QGC model to assess the change in the incidence of sleep problems when 15 VOC metabolites were simultaneously elevated by a quarter. The QGC model, combined with the adaptability of g calculations, can be used to assess the cumulative effects of multiple pollutants in different directions [27].

All statistical analyses were performed using R version 4.1.0 (Bioconductor, NY, USA). Weighted generalized logistic regression, RCS, WQS, and QGC analyses were performed using the “survey” package, “rms” package, “gwqs” package, and “qgcomp” package, respectively [27]. *p* values of <0.05 were considered statistically significant.

## 3. Results

### 3.1. General Characteristics of the Included Population

As shown in Table 2, all participants were categorized into SSD or non-SSD subgroups and trouble-sleeping or non-trouble-sleeping subgroups. A total of 4131 participants were included, including 1409 with SSD and 1034 with trouble sleeping. The SSD and non-SSD subgroups were statistically different in terms of PIR, serum cotinine, creatinine, gender, race, and education. Statistically significant differences existed between the trouble-sleeping and non-trouble-sleeping groups with respect to age, BMI, gender, race, marital status, physical activity, and alcohol consumption. SSD and trouble sleeping were more prevalent among the non-Hispanic white and non-Hispanic black groups.

As shown in Table 3, the remaining 12 VOCs were higher in SSD participants than in non-SSD participants, except for three VOCs, AMCC, ATCA, and 2HPMA. For the trouble-sleeping and non-trouble-sleeping subgroups, AMCC, CEMA, CYMA, MHBMA3, and HPMMA were higher in the urine of trouble-sleeping participants than that of non-trouble-sleeping participants, and there were no statistically significant differences in the other VOCs.

### 3.2. Association between Each Kind of VOC and Sleep Problems

After adjusting for covariates, 3,4-MHA, AAMA, AMCC, SBMA, and MA were risk factors for SSD with odds ratios (ORs) of 1.27 (95% CI: 1.03, 1.57), 1.44 (95% CI: 1.04, 1.98), 1.47 (95% CI: 1.08, 2.02), 1.30 (95% CI: 1.02, 1.64), and 1.38 (95% CI: 1.91, 1.00), with the largest effect being caused by AMCC. AAMA, AMCC, CEMA, CYMA, DGBMA, 2HPMA, 3HPMA, MA, and PGA were the risk factors for trouble sleeping, with ORs of 1.49 (95% CI: 1.06, 2.11), 1.69 (95% CI: 1.28, 2.22), 1.51 (95% CI: 1.14, 1.98), 1.33 (95% CI: 1.16, 1.54), 1.66 (95% CI: 1.07, 2.59), 1.44 (95% CI: 1.14, 1.82), 1.56 (95% CI: 1.13, 2.16), 1.44 (95% CI: 1.16, 1.79), and 1.41 (95% CI: 1.11, 1.78), respectively, with the largest impact being caused by AMCC (Table 4).

**Figure 1:**
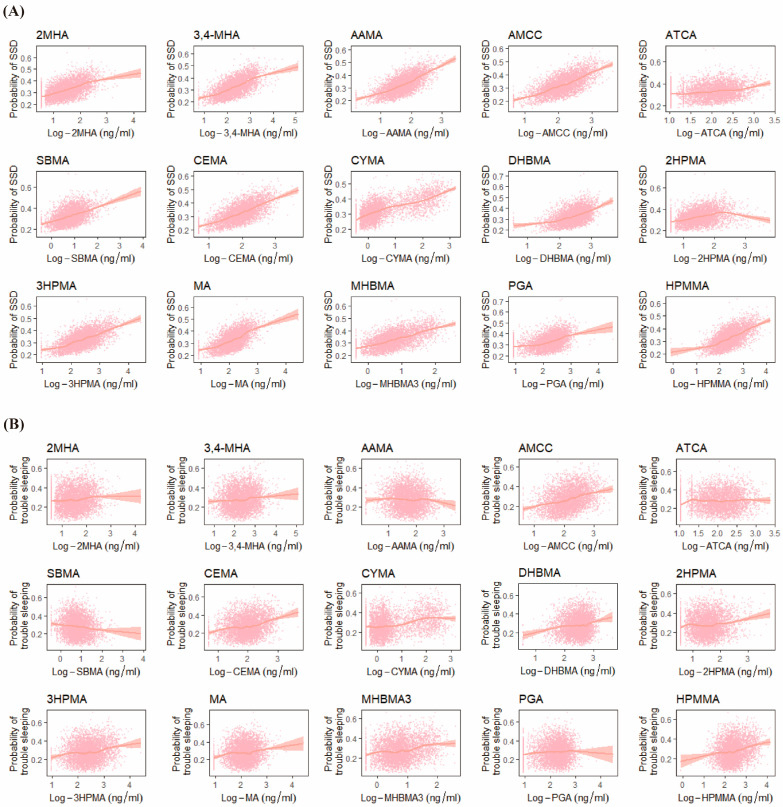
Relationship between VOCs and SSD (**A**) and trouble sleeping (**B**). The scatter region is the probability distribution of the variables corresponding to the X-axis. Adjusted for creatinine, sex, age, educational level, race, marriage, PIR, BMI, serum cotinine, drinking alcohol, and physical activity.

According to the results of the RCS (Figure 1), the metabolites of 14 VOCs were positively associated with SSD, except for 2HPMA. AMCC, CEMA, 2HPMA, 3HPMA, MA, and HPMA were positively associated with trouble sleeping.

### 3.3. Association between Co-Exposure to VOCs and Sleep Problems

**Figure 2:**
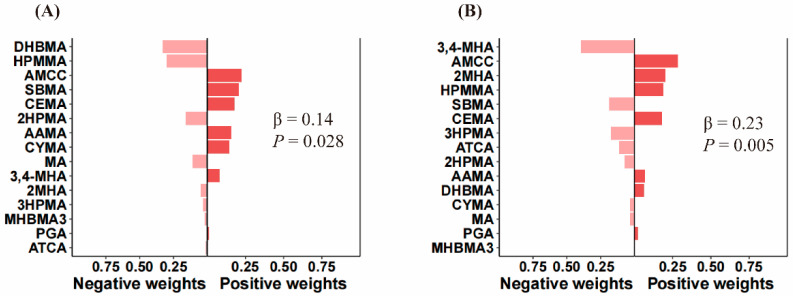
Estimated relationship and weight between combined exposure to VOCs and SSD (**A**) and trouble sleeping (**B**) by QGC models. VOCs were log-transformed. The model was adjusted for creatinine, sex, age, educational level, race, marriage, PIR, BMI, serum cotinine, drinking alcohol, and physical activity.

**Figure 3:**
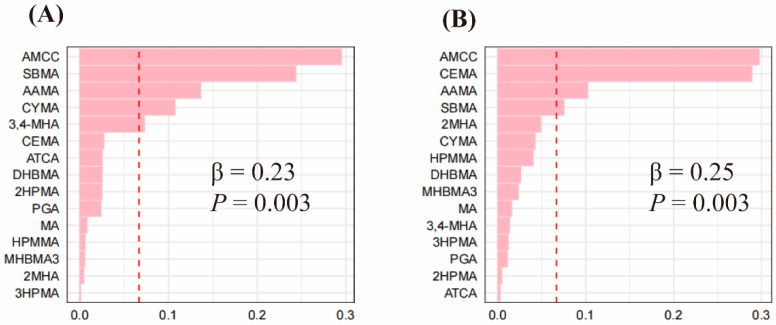
Estimated relationship and weighted values of VOCs for SSD (**A**) and trouble sleeping (**B**) by WQS models. VOCs were log-transformed. The model was adjusted for creatinine, sex, age, educational level, race, marriage, PIR, BMI, serum cotinine, drinking alcohol, and physical activity.

The QGC results showed that co-exposure to VOC metabolites was positively associated with SSD (β = 0.14, *p* = 0.028) and trouble sleeping (β = 0.23, *p* = 0.005), with AMCC having the largest positive weights of 0.25 and 0.32, respectively (Figure 2). The results of WQS modeling similarly showed that co-exposure to VOC metabolites was positively associated with SSD (β= 0.23, *p* = 0.003) and trouble sleeping (β = 0.25, *p* = 0.003). The VOC with the greatest impact on the association of combined exposure to VOCs with SSD was AMCC (weight = 0.29), followed by SBMA (weight = 0.24) and AAMA (weight = 0.14). The VOC with the greatest influence on the relationship between combined exposure to VOCs and trouble sleeping was also AMCC (weight = 0.30), followed by CEMA (weight = 0.29) and AAMA (weight = 0.10) (Figure 3).

## 4. Discussion

In this study, we investigated the relationship between sleep problems and urine VOC metabolites using the NHANES dataset. A total of 4131 subjects were included in the study and analyzed using 15 metabolites of VOCs in urine. The results of the study showed that among the 15 VOCs, some of the VOC metabolites were positively associated with SSD and most of the VOC metabolites were positively associated with trouble sleeping. Furthermore, we confirmed that co-exposure to VOCs resulted in a significant increase in the prevalence of SSD and trouble sleeping in adults by the WQS model and QGC model analysis.

This study found that, among the general adult population in the United States, exposure to VOCs was associated with SSD and trouble sleeping. Several previous studies have also reported associations between environmental pollutants and sleep health. Some studies have found that exposure to metal fumes was associated with sleep disorders in shipyard welders [28]. Exposure to cooking oil fumes from home cooking was associated with poor sleep quality in middle-aged Chinese people [29]. And one study found that children exposed to conventional biomass stoves had a higher frequency of sleep apnea symptoms [30]. Pollutants in the environment are generally thought to influence sleep outcomes through changes in central nervous system regulation and/or respiratory physiology [31]. First, VOCs may have a direct effect on the central nervous system, resulting in altered and dysregulated neurochemical expression [32]. Specifically, the penetration of VOCs into the brain alters serotonin levels [28], disrupts the protective epithelial barrier, and destroys nerve cells [31,33]. These alterations may interfere with brain function and cause sleep problems. Second, VOCs may harm respiratory cells, leading to inflammation, infections, or the greater restriction and obstruction of airflow, resulting in respiratory-related sleep problems and poor sleep quality [31]. Furthermore, VOCs affect the human endocrine system, causing an endocrine disturbance [34]. The endocrine system is closely associated with sleep problems, and abnormal hormonal homeostasis may significantly affect sleep patterns [35]. One study found that phthalates alter brain circuits and hinder the maturation of hormone-mediated systems, causing sleep difficulties in adolescents [11]. Another study discovered that bisphenol A may impair upper airway muscle function, resulting in obstructive sleep apnea [36].

In essence, RCS fits the spline function RCS by selecting the position and number of nodes, so that the continuous variable presents a smooth curve in the whole value range to realize the exploration of nonlinear relations [37]. People are often exposed to multiple VOCs, and it is more than imperative to explore the effects of exposure to a mixture of VOCs on sleep. We used both the WQS model and the QCG model to quantify and visualize the effects on sleep when mixtures of VOCs are co-exposed. Both models could assess the contribution of each pollutant to the outcome in mixed exposure. The WQS model consisted of 1000 bootstrap samples per group, divided into training (40%) and verification (60%) sets, without limiting the direction of association between mixture and outcome [12]. However, due to the assumption of direction consistency, there may be some deviation in the model estimation results. At the same time, the WQS model defaults to linear correlation between variables and outcomes and addition between variables, but most studies do not support this assumption [38]. To address the above limitations, we employed the QGC model, which combines the simple reasoning of the WQS regression model with the adaptability of g calculations to evaluate the cumulative effects of multiple variable chemicals in different directions [27]. However, it must be noted that the length of the QGC weight bar chart could only be compared with each other in the same direction, and the left and right sides of the chart could not be compared with each other [27]. Darker colors on the right side indicate that mixed exposure to VOCs is positively associated with sleep problems.

We found that AMCC was the risk factor with the greatest positive weight for SSD and trouble sleeping. AMCC is a dimethylformamide (DMF) metabolite. DFM is an excellent solvent for acetylene extraction and the production of polyacrylonitrile fibers, which are widely utilized in the industries of imitation leather, organic synthesis, dyestuffs, medicines, petroleum refining, and resins. Previous research has revealed that DFM causes liver damage, renal damage, lung damage, brain damage, and immunological dysfunction [39]. We found that AMCC, a key metabolite of DMF, was connected with sleep problems in this study, and we concluded that DMF is a possible risk factor for SSD and trouble sleeping. DFM, like other environmental contaminants, may cause sleep disorders by affecting the respiratory and neurological systems. However, the particular physiological pathways that relate DFM and AMCC to sleep problems are unknown, and more in vivo research is required to investigate the toxicity mechanisms.

Apart from AMCC, CEMA has the largest influence on sleep problems. Acrolein is CEMA’s parent chemical. Acrolein has numerous applications, including diapers, acrylate polymers, paints, and coatings, as well as bactericides in the pharmaceutical, water treatment, and petroleum industries [40]. Acrolein metabolism in the human body remains unclear. In rats, it is first transformed to acrylic acid by the creation of methyl esters, then to 3HPMA via glutathione binding, and it is ultimately oxidized to generate CEMA and excreted in urine [41]. Our results found that 3HPMA as an intermediate product also had a significant effect on sleep problems. However, as acrolein levels rise in the body, so does the conversion of 3HPMA to CEMA, and urine CEMA may be a better biomarker of acrolein exposure levels than 3HPMA [40]. Excessive acrolein exposure may be a risk factor for trouble sleeping, but CEMA and 3HPMA have not been linked to SSD. By triggering the release of peptides in innervating nerve terminals, acrolein can cause respiratory, ocular, and gastrointestinal discomfort [41]. Acrolein exposure causes increased apoptosis in alveolar macrophages, increased mucus secretion, increased pulmonary edema, and enhanced bronchial reactivity, according to toxicological investigations [41]. Acrolein may contribute to trouble sleeping through these mechanisms.

AAMA and MA were also found to be positively linked with SSD and sleep issues. Acrylamide and styrene are the parent chemicals of AAMA and MA, respectively. Acrylamide is the raw ingredient needed to make polyacrylamide, which is mostly utilized in water purification and treatment, pulp processing, and pipe internal coating. Acrylamide and its metabolite, glycidylamine, have neurotoxic, genotoxic, and carcinogenic qualities, affecting peripheral nerve signaling, enzymatic and hormonal control, muscle function, reproduction, and other processes [42]. Styrene monomer is widely used in the plastic and synthetic rubber industries, and clinical studies have shown that styrene is toxic to both the central and peripheral nervous system, and chronic exposure to styrene leads to alterations in neurobehavioral and neurophysiological measures [43]. AAMA and MA may lead to sleep problems by damaging the nervous system, but further studies are needed to clarify the specific physiological mechanisms.

To prevent sleep problems, the use of cleaning products containing VOCs should be avoided at home, in hospitals, or in nursing homes. Healthcare workers should avoid releasing volatile VOCs after storing or using perfume. There are still several limitations in this study. First, since this research design was cross-sectional, it was unable to determine the cause-and-effect relationship between exposure to VOCs and sleep issues. More cohort and experimental studies are needed in the future to establish a causal relationship between the two. Second, because the metabolic processes of many VOCs in the body are not well understood, further research needs to be carried out on the accuracy of using some single VOCs in urine to indicate relative VOC exposure. However, the direct detection of VOCs in breath and blood does not always yield accurate results due to the volatility of VOCs [44]. VOCs in urine have a more stable and longer biological half-life than VOCs in blood [45]. Therefore, identifying VOCs in urine is thought to yield more reliable test findings [23]. Future studies could benefit from direct measures of environmental VOC concentrations. Third, because a considerable fraction of the eligible study population lacked covariate data, there could be selection bias. Fourth, while the study adjusted for several covariates, there may be unmeasured factors (e.g., occupational exposures, indoor air quality) that could have confounded the observed associations. Additionally, measurement errors may have arisen due to, for example, not using standard tools like the Chicago Screening interview or similar to determine the presence of sleep disorders. These questionnaires measure not only the time to fall asleep, but also the latency period from sleep to sleep, the number of times one wakes up during the night, and subjective feelings of fatigue or rest after sleep. It is also highly recommended to use objective measurement methods such as actigraphs to improve the accuracy of studies in the future. Finally, given the study’s focus on the US adult population, the findings may not be directly applicable to other populations or age groups. Studies in diverse settings are warranted.

## 5. Conclusions

We analyzed the association between urinary VOC metabolites and SSD and sleep difficulties using the NHANES dataset. Our findings found that 3,4-MHA, AAMA, AMCC, SBMA, and MA are risk factors for SSD; AAMA, AMCC, CEMA, CYMA, DGBMA, 2HPMA, 3HPMA, MA, and PGA are risk factors for sleep difficulties. Mixed urinary VOC metabolite levels were associated with the increased prevalence of SSD and trouble sleeping in the general adult population in the US, with AMCC having the greatest impact on this relationship. This has important implications for preventing VOC pollution and protecting sleep health. Further prospective and experimental studies are needed in the future to validate these potential relationships and explore the underlying mechanisms.

## Figures and Tables

**Table 1:** VOC metabolites included in the study and corresponding abbreviations.

VOC Metabolites	Abbreviation
2-methylhippuric acid	2MHA
3- and 4-methylhippuric acid	3,4-MHA
N-acetyl-S-(2-carbamoylethyl)-L-cysteine	AAMA
N-acetyl-S-(N-methylcarbamoyl)-L-cysteine	AMCC
2-aminothiazoline-4-carboxylic acid	ATCA
N-acetyl-S-(benzyl)-L-cysteine	SBMA
N-acetyl-S-(2-carboxyethyl)-L-cysteine	CEMA
N-acetyl-S-(2-cyanoethyl)-L-cysteine	CYMA
N-acetyl-S-(3,4-dihydroxybutyl)-L-cysteine	DHBMA
N-acetyl-S-(2-hydroxypropyl)-L-cysteine	2HPMA
N-acetyl-S-(3-hydroxypropyl)-L-cysteine	3HPMA
Mandelic acid	MA
N-acetyl-S-(4-hydroxy-2-butenyl)-L-cysteine	MHBMA3
Phenylglyoxylic acid	PGA
N-acetyl-S-(3-hydroxypropyl-1-methyl)-L-cysteine	HPMMA

**Table 2:** Baseline data of included participants.

Baseline Data	Non-SSD (n = 2722)	SSD (n = 1409)	*p* Value	Non-Trouble Sleeping (n = 3097)	Trouble Sleeping (n = 1034)	*p* Value
Age (year, SD)	47.10 (0.63)	47.93 (0.56)	0.219	46.20 (0.58)	51.47 (0.67)	<0.001 †,**
BMI (kg/m^2^, SD)	28.63 (0.24)	28.16 (0.16)	0.062	28.02 (0.16)	29.06 (0.22)	<0.001 †,**
PIR (SD)	2.97 (0.07)	3.18 (0.06)	0.014 *	3.10 (0.05)	3.14 (0.07)	0.595 †
Serum cotinine (ng/mL, SD)	73.39 (5.61)	52.49 (3.26)	0.001 **	56.15 (3.56)	66.79 (5.76)	0.129 ‡
Creatinine (μg/mL, SD)	118.26 (2.52)	109.36 (2.04)	0.006 **	113.94 (2.09)	107.62 (3.01)	0.108 ‡
Gender						
Male	1349 (49.56)	776 (55.07)	<0.001 **	1639 (52.92)	486 (47.00)	0.016 *
Female	1373 (50.44)	633 (44.93)		1458 (47.08)	548 (53.00)	
Race						
Mexican American	470 (17.27)	212 (15.05)	<0.001 **	571 (18.44)	111 (10.74)	<0.001 **
Other Hispanic	194 (7.13)	111 (7.88)		234 (7.56)	71 (6.87)	
Non-Hispanic white	1269 (46.62)	534 (37.90)		1256 (40.56)	547 (52.90)	
Non-Hispanic black	466 (17.12)	419 (29.74)		675 (21.80)	210 (20.31)	
Other	323 (11.87)	133 (9.44)		361 (11.66)	95 (9.19)	
Educational level						
Less than 9th grade	284 (10.43)	114 (8.09)	<0.001 **	327 (10.56)	71 (6.87)	0.105
9–11th grade	331 (12.16)	209 (14.83)		415 (13.40)	125 (12.09)	
High school graduate	613 (22.52)	324 (23.00)		691 (22.31)	246 (23.79)	
Some college or AA degree	738 (27.11)	453 (32.15)		839 (27.09)	352 (34.04)	
College graduate or above	756 (27.77)	309 (21.93)		825 (26.64)	240 (23.21)	
Marital status						
Married	1530 (56.21)	687 (48.76)	0.098	1684 (119.52)	533 (37.83)	<0.001 **
Widowed	184 (6.76)	90 (6.39)		197 (13.98)	77 9 (5.46)	
Divorced	264 (9.70)	185 (13.13)		289 (20.51)	160 (11.36)	
Separated	65 (2.39)	50 (3.55)		75 (5.32)	40 (2.84)	
Never married	448 (16.46)	263 (18.67)		568 (40.31)	143 (10.15)	
Living with partner	231 (8.49)	134 (9.51)		284 (20.16)	81 (5.75)	
Body activity						
None	1004 (36.88)	517 (36.69)	0.785	1179 (38.07)	342 (33.08)	0.016 *
Moderate	836 (30.71)	422 (29.95)		924 (29.84)	334 (32.30)	
Vigorous	882 (32.40)	470 (33.36)		994 (32.10)	358 (34.62)	
Drinking alcohol						
Yes	2043	1048	0.664	2264	827	<0.001 **
No	679	361		833	207	

SSD, short sleep duration; SD, standard deviation; BMI, body mass index; PIR, the ratio of family income to poverty. Student’s *t*-test or Kruskal–Wallis tests were used for the comparison of the continuous variables according to the data distribution and the Chi-square test for the categorical variables. † Student’s *t*-test, ‡ Kruskal–Wallis ** *p* < 0.01; * *p* < 0.05.

**Table 3:** The concentrations of volatile organic compound metabolites (VOCs) in the urine of non-SSD and SSD subgroups, as well as non-trouble-sleeping and trouble-sleeping subgroups.

VOCs (ng/mL)	Non-SSD (n = 2722)	SSD (n = 1409)	*p* Value	Non-Trouble Sleeping (n = 3097)	Trouble Sleeping (n = 1034)	*p* Value
2MHA	27.80 (12.60, 72.40)	35.6 (16.5, 88.5)	<0.001 **	28.9 (13.2, 73.8)	33.7 (13.3, 83.6)	0.289
3,4-MHA	180.0 (77.2, 488.0)	242.0 (104.0, 592.0)	<0.001 **	202.0 (81.7, 504.0)	207.0 (89.3, 547.0)	0.369
AAMA	47.2 (24.7, 93.1)	58.0 (29.2, 120.0)	<0.001 **	49.9 (25.7, 99.6)	53.2 (27.2, 102.0)	0.407
AMCC	139.0 (66.9, 294.0)	174.0 (88.2, 371.0)	0.061	139.0 (67.8, 297.0)	185.0 (87.8, 363.0)	<0.001 **
ATCA	90.3 (36.6, 193.0)	104.0 (42.4, 201.0)	0.128	94.2 (36.4, 197.0)	99.8 (44.7, 193.0)	0.300
SBMA	5.8 (2.9, 11.3)	6.7 (3.5, 12.4)	0.002 **	6.1 (3.2, 11.9)	5.9 (3.0, 11.1)	0.273
CEMA	86.9 (41.2, 163.0)	106.0 (51.6, 199.0)	<0.001 **	88.9 (41.6, 168.0)	98.9 (49.0, 190.0)	0.049 *
CYMA	1.8 (0.9, 5.9)	1.9 (1.2, 25.5)	<0.001 **	1.8 (0.9, 5.7)	1.8 (1.0, 22.8)	0.006 **
DHBMA	281.0 (152.0, 477.0)	325.0 (178.0, 518.0)	<0.001 **	293.0 (154.0, 491.0)	301.0 (174.0, 493.0)	0.329
2HPMA	29.7 (14.6, 61.3)	34.7 (17.4, 67.2)	0.016	31.0 (15.4, 62.0)	33.4 (15.6, 63.4)	0.465
3HPMA	223.0 (108.0, 451.0)	254.0 (130.0, 581.0)	<0.001 **	234.0 (112.0, 465.0)	235.0 (116.0, 562.0)	0.133
MA	127.0 (68.2, 222.0)	150.0 (78.1, 263.0)	<0.001 **	132.0 (70.7, 233.0)	133.0 (72.8, 241.0)	0.313
MHBMA3	4.77 (2.33, 10.60)	5.7 (2.8, 14.3)	<0.001 **	4.8 (2.4, 10.6)	5.5 (2.7, 13.7)	0.018 *
PGA	174.0 (78.9, 317.0)	201.0 (97.1, 355.0)	<0.001 **	178.0 (81.5, 331.0)	187.0 (95.2, 326.0)	0.547
HPMMA	216.0 (108.0, 443.0)	247.0 (132.0, 523.0)	<0.001 **	219.0 (113.0, 446.0)	260.0 (126.0, 516.0)	0.035 *

VOC metabolite distribution data in urine are expressed as median (interquartile). ** *p* < 0.01; * *p* < 0.05.

**Table 4:** Associations of VOC concentrations with SSD and trouble sleeping in generalized linear regression models.

	VOCs	Model 1		Model 2	
		OR (95% CI)	*p* Value	OR (95% CI)	*p* Value
SSD	2MHA	1.39 (1.17, 1.64)	<0.001 **	1.17 (0.95, 1.45)	0.136
	3,4-MHA	1.44 (1.24, 1.68)	<0.001 **	1.27 (1.03, 1.57)	0.027 *
	AAMA	1.63 (1.33, 2.01)	<0.001 **	1.44 (1.04, 1.98)	0.026 *
	AMCC	1.58 (1.29, 1.93)	<0.001 **	1.47 (1.08, 2.02)	0.016 *
	ATCA	1.14 (0.98, 1.33)	0.083	1.12 (0.95, 1.33)	0.177
	SBMA	1.37 (1.13, 1.66)	0.001 **	1.30 (1.02, 1.64)	0.032 *
	CEMA	1.56 (1.29, 1.89)	<0.001 **	1.30 (0.97, 1.74)	0.081
	CYMA	1.25 (1.13, 1.38)	<0.001 **	1.13 (0.97, 1.32)	0.118
	DHBMA	1.52 (1.21, 1.91)	<0.001 **	1.17 (0.76, 1.80)	0.471
	2HPMA	1.22 (1.01, 1.48)	0.037 *	0.99 (0.77, 1.27)	0.951
	3HPMA	1.45 (1.21, 1.73)	<0.001 **	1.14 (0.89, 1.46)	0.283
	MA	1.61 (1.30, 1.99)	<0.001 **	1.38 (1.91, 1.00)	0.048 *
	MHBMA3	1.39 (1.18, 1.63)	<0.001 **	1.11 (0.88, 1.41)	0.379
	PGA	1.32 (1.12, 1.55)	0.001 *	1.08 (0.89, 1.31)	0.458
	HPMMA	1.46 (1.24, 1.72)	<0.001 **	1.19 (0.94, 1.50)	0.158
Trouble sleeping	2MHA	1.11 (0.90, 1.37)	0.323	1.21 (0.95, 1.55)	0.124
	3,4-MHA	1.11 (0.91, 1.35)	0.296	1.22 (0.96, 1.54)	0.100
	AAMA	1.11 (0.86, 1.42)	0.434	1.49 (1.06, 2.11)	0.022 *
	AMCC	1.45 (1.17, 1.80)	<0.001 **	1.69 (1.28, 2.220	<0.001 **
	ATCA	1.14 (0.94, 1.39)	0.175	1.12 (0.90, 1.39)	0.317
	SBMA	0.86 (0.70, 1.08)	0.191	0.83 (0.65, 1.05)	0.119
	CEMA	1.28 (1.01, 1.63)	0.039	1.51 (1.14, 1.98)	0.004 **
	CYMA	1.19 (1.05, 1.34)	0.005 **	1.33 (1.16, 1.54)	<0.001 **
	DHBMA	1.17 (0.87, 1.55)	0.298	1.66 (1.07, 2.59)	0.025 *
	2HPMA	1.19 (0.96, 1.48)	0.120	1.44 (1.14, 1.82)	0.002 **
	3HPMA	1.15 (0.89, 1.49)	0.271	1.56 (1.13, 2.16)	0.007 **
	MA	1.23 (1.02, 1.49)	0.032 *	1.44 (1.16, 1.79)	0.001 **
	MHBMA3	1.07 (0.88, 1.30)	0.478	1.09 (0.88, 1.36)	0.518
	PGA	1.25 (1.01, 1.55)	0.042 *	1.41 (1.11, 1.78)	0.005 **
	HPMMA	1.25 (1.01, 1.55)	0.042 *	1.41 (1.11, 1.78)	0.583

SSD, short sleep duration; VOCs were log-transformed; OR > 1 means positive correlation, and OR < 1 means negative correlation. Model 1: no adjustment; Model 2: adjusted for creatinine, sex, age, educational level, race, marriage, PIR, BMI, serum cotinine, drinking alcohol, and physical activity. ** *p* < 0.01; * *p* < 0.05.

## Data Availability

All data are available from open access websites at http://ghdx.healthdata.org/gbd-results-tool (accessed on 20 August 2023).

## Supplementary Materials

The following supporting information can be downloaded at: https://www.mdpi.com/article/10.3390/toxics12030222/s1, Figure S1: The flow gram of screening out eligible participants from six survey cycles (2005–2006, 2011–2012, 2013–2014, 2015–2016, 2017–2018) of the National Health and Nutrition Examination Survey (NHANES) program.
