# Supplementary material for: Association between Exposure to Volatile Organic Compounds and the Prevalence of Sleep Problems in US Adults
Source: Toxics. 2024 Mar 18;12(3):222. doi: 10.3390/toxics12030222 (PMC10976002; doi:10.3390/toxics12030222)
Supplement: Supplementary file 1 [file toxics-12-00222-s001.zip › toxics-2891779-supplementary.pdf]

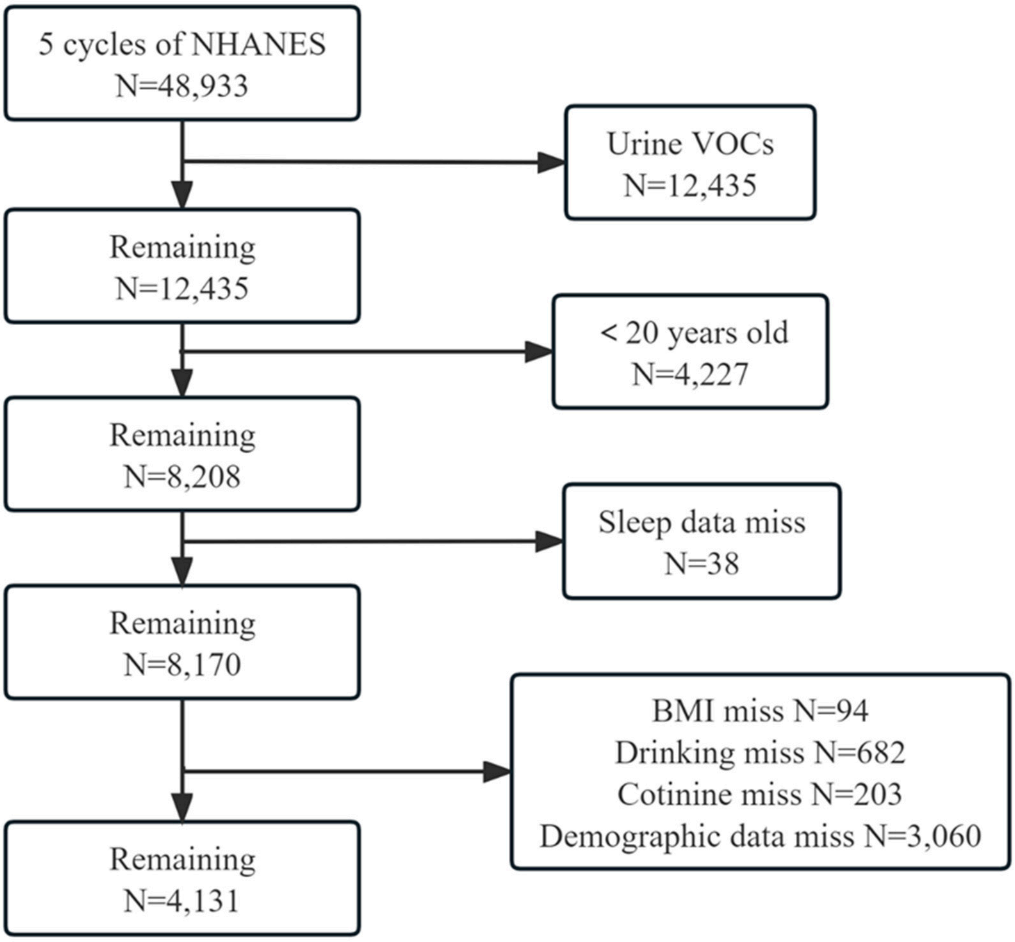

Figure. S1 The flow gram of screening out eligible participants from 6 survey cycles (2005–2006, 2011–2012, 2013–2014, 2015–2016, 2017–2018) of the National Health and Nutrition Examination Survey (NHANES) program.
